# Giant Cell Arteritis Mimicking Polymyalgia Rheumatica: A Challenging Diagnosis

**DOI:** 10.7759/cureus.27517

**Published:** 2022-07-31

**Authors:** Ryuichi Ohta, Tatsuhiko Okayasu, Noritaka Katagiri, Takafumi Yamane, Minami Obata, Chiaki Sano

**Affiliations:** 1 Communiy Care, Unnan City Hospital, Unnan, JPN; 2 Family Medicine, International University of Health and Welfare, Tokyo, JPN; 3 Family Medicine, Shimane Universtity, Izumo, JPN; 4 Family Medicine, Yamane Clinic, Unnan, JPN; 5 Communiy Care, Unnan CIity Hospital, Unnan, JPN; 6 Community Medicine Management, Shimane University Faculty of Medicine, Izumo, JPN

**Keywords:** general physician, rural hospitals, older, polymyalgia rheumatica, giant cell arteritis

## Abstract

Giant cell arteritis (GCA) is an autoimmune disease that causes inflammation of the middle and large arteries. Rural areas have many older patients with various symptoms, so large-vessel-type GCA should be managed effectively. Older patients tend to show vague symptoms that cannot be adequately diagnosed and observed. Here, we have encountered a case of a 91-year-old woman with a chief complaint of fatigue diagnosed with large-vessel type GCA in collaboration with a rural clinic. Effective collaboration between physicians in rural hospitals and clinics is necessary for diagnosing and treating large-vessel GCA. In rural areas, without adequate healthcare professionals, physicians should share their abilities and collaborate smoothly to mitigate delays in consultation and treatment. To effectively treat large vessel-type GCA, rural general physicians should be familiar with the clinical course of the disease and treatment for rural comprehensive care.

## Introduction

Giant cell arteritis (GCA) is an autoimmune disease that causes inflammation of the middle and large arteries [1]. Despite its previous nomenclature as temporal arteritis, the disease is prevalent in the temporal artery and middle-to-large arteries [2]. Typical symptoms include fever, headache, and jaw claudication caused by peripheral ischemia; however, GCA involving large vessels commonly presents with unspecific symptoms like fatigue and fever, making it a diagnostic challenge [1,3,4]. GCA can affect the arteries of the eyes and produce pericardial lesions; therefore, prompt diagnosis and treatment should be undertaken. Depending on the severity of GCA steroids, immunosuppressants or biologics are chosen as treatment options [4].

As rural areas have many older patients with various symptoms, large-vessel-type GCA should be managed effectively through collaboration with various medical professionals. Older patients tend to exhibit vague symptoms that cannot be appropriately diagnosed and observed [5,6]. Older people frequently tend to have various vague symptoms, so the differential diagnosis may not initially include GCA. However, a delayed diagnosis of GCA can result in aortic dissection and blindness [3]. The symptoms of GCA may be similar to polymyalgia rheumatica (PMR) [4]. PMR can be treated with a small dose of steroids; however, GCA requires high-dose steroids with immunosuppressants or biologics such as IL6 inhibitors [7]. Rural clinics and hospitals should collaborate to diagnose GCA in rural contexts effectively. The present case of a large-vessel GCA was diagnosed in collaboration with a rural clinic. This case highlights the importance of identifying the difference between PMR and GCA and the need for smooth collaboration between rural healthcare professionals and patients.

## Case presentation

A 91-year-old woman presented at a community hospital with complaints of general malaise and fever. Six months prior, the patient experienced dull pain in both the lower and upper limbs and experienced difficulty getting up. She did not report any headache, eyesight impairment, or jaw claudication. Her primary care physician treated her with 20 mg of prednisolone based on a diagnosis of PMR four months ago. Subsequently, when the prednisolone dose was tapered to 5 mg one month ago, she experienced a recurrence of general malaise and fatigue in her lower limbs. Four days before admission, her blood test revealed a C-reactive protein (CRP) level of 15.85 mg/dL, and she could not move by herself. The patient was referred to our hospital for further evaluation. Her medical history included hypertension. She was receiving tizanidine, Prostigmine, celecoxib, lemborexant, and prednisolone as medications.

On admission, her vital signs were as follows: body temperature, 36.9°C; pulse rate, 106/min; respiration rate, 16 times/min; blood pressure, 145/74 mmHg; and SpO_2_, 98% at room air. She complained of shoulder and thigh pain without visual abnormalities or tenderness of the temporal arteries. Vascular murmurs were observed in both clavicular fossae. Physical examination of the chest, abdomen, and joints revealed no abnormalities. Blood tests revealed an erythrocyte sedimentation rate of 84 mm/h, CRP level of 13.14 mg/dl, a ferritin level of 380.3 ng/ml, and urinalysis revealed leukocytes (1+), protein (1+), and occult blood (±) (Table 1).

**Table 1:** Initial laboratory data of the patient PT, Prothrombin time; INR, International normalized ratio; APTT, Activated partial thromboplastin time; eGFR, Estimated glomerular filtration rate; CK, Creatine kinase; CRP, C-reactive protein; TSH, Thyroid-stimulating hormone; Ig, Immunoglobulin; HCV, Hepatitis C virus; SARS-CoV-2, Severe acute respiratory syndrome coronavirus 2; HIV, Human immunodeficiency virus; HBs, Hepatitis B surface antigen; HBc, Hepatitis B core antigen; C3, Complement component 3; C4, Complement component 4; MPO-ANCA, Myeloperoxidase antibody proteinase 3 antibodies; SS, Sjögren's syndrome; CCP, Cyclic citrullinated peptide.

Marker	Level	Reference
White blood cells	9.40	3.5–9.1 × 10^3^/μL
Neutrophils	72.3	44.0%–72.0%
Lymphocytes	11.3	18.0%–59.0%
Monocytes	15.1	0.0%–12.0%
Eosinophils	0.4	0.0%–10.0%
Basophils	0.9	0.0%–3.0%
Red blood cells	3.66	3.76–5.50 × 10^6^/μL
Hemoglobin	11.3	11.3–15.2 g/dL
Hematocrit	34.2	33.4%–44.9%
Mean corpuscular volume	93.6	79.0–100.0 fl
Platelets	33.3	13.0–36.9 × 10^4^/μL
Erythrocyte sedimentation rate	84	2–10 mm/hour
Total protein	6.7	6.5–8.3 g/dL
Albumin	3.1	3.8–5.3 g/dL
Total bilirubin	0.7	0.2–1.2 mg/dL
Aspartate aminotransferase	18	8–38 IU/L
Alanine aminotransferase	16	4–43 IU/L
Alkaline phosphatase	83	106–322 U/L
γ-Glutamyl transpeptidase	27	<48 IU/L
Lactate dehydrogenase	161	121–245 U/L
Blood urea nitrogen	10.7	8–20 mg/dL
Creatinine	0.76	0.40–1.10 mg/dL
eGFR	53.0	>60.0 mL/min/L
Serum Na	133	135–150 mEq/L
Serum K	4.2	3.5–5.3 mEq/L
Serum Cl	100	98–110 mEq/L
Serum Ca	9.9	3.5–5.3 mg/dL
Serum P	3.8	0.2–1.2 mg/dL
Serum Mg	1.9	1.8–2.3 mg/dL
Ferritin	380.3	14.4–303.7 ng/mL
CK	22	56–244 U/L
CRP	13.14	<0.30 mg/dL
TSH	1.10	0.35–4.94 μIU/mL
Free T4	1.2	0.70–1.48 ng/dL
IgG	1442	870–1700 mg/dL
IgM	41	35–220 mg/dL
IgA	282	110–410 mg/dL
IgE	17	<173 mg/dL
HBs antigen	0.0	IU/mL
HBs antibody	0.67	mIU/mL
HBc antibody	0.00	S/CO
HCV antibody	0.00	S/CO
Syphilis treponema antibody	0.00	S/CO
SARS-CoV-2 antigen	-	
Anti-nuclear antibody	40	<40
Homogeneous	40	<40
Speckled	40	<40
C3	164	86–164 mg/dl
C4	33	17–45 mg/dl
MPO-ANCA	<1.0	<3.5 U/ml
Anti-SS-A/Ro antibody	<1.0	<10.0 U/ml
Anti-SS-B/La antibody	<1.0	<10.0 U/ml
Anti-CCP antibody	<0.6	<5 U/ml
Anti-cardiolipin antibody IgG	8.2	<12.3 U/ml
Urine test		
Leukocyte	(1+)	
Nitrite	(-)	
Protein	(1+)	
Glucose	(-)	
Urobilinogen	Normal	
Bilirubin	(-)	
Ketone	(-)	
Blood	(+-)	
pH	6.5	
Specific gravity	1.013	

**Figure 1:**
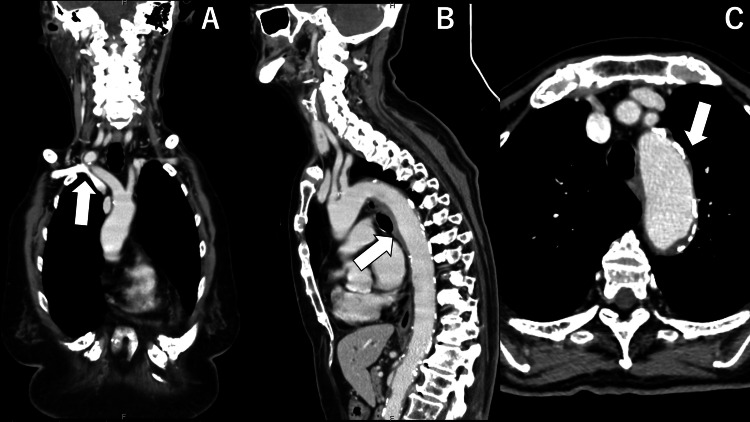
CT chest angiography, showing enhanced aortic wall from ascending to descending parts and stricture of the descending aorta and subclavian arteries (white arrows): (A) coronal, (B) sagittal, and (C) transverse views

We performed a blood culture, suspecting a bloodstream infection due to steroid administration, resulting in no growth. At this moment, GCA was considered a differential diagnosis. Contrast-enhanced chest CT showed the wall thickening and a contrast enhancement from the aorta to the subclavian artery (Figure 1).

**Figure 2:**
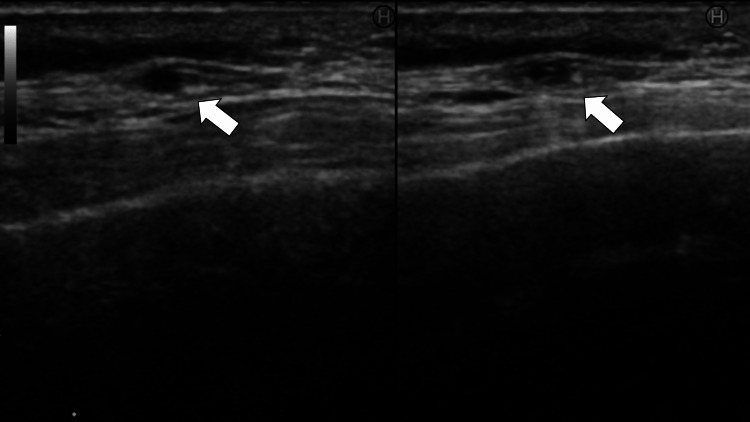
Ultrasound examination on temporal artery revealing halo signs in the compression on the artery (white arrows)

Ultrasound examination of the temporal arteries revealed bilateral temporal halo signs (Figure 2).

Based on these findings, her condition was diagnosed as giant cell vasculitis, and she was admitted to the hospital for treatment.

Giant cell vasculitis was treated using prednisolone (1 mg/kg/day) and tocilizumab (6 mg/kg) monthly. The general malaise and shoulder and thigh tenderness resolved on the third day of treatment. Blood tests showed the absence of obvious cytopenia and resolution of inflammatory markers. We considered tocilizumab effective and tapered prednisolone to 10 mg every three days from the eighth day. The patient’s condition was without any exacerbation. The patient was discharged on the 14th day after admission. In the outpatient department, her prednisolone dose was tapered to zero on the 30th day after admission, with the continuation of monthly tocilizumab.

## Discussion

To effectively diagnose GCA, the clinical courses of GCA and PMR should be considered in the continual spectrum. Typically, GCA shows temporal headaches because of temporal artery inflammation; therefore, it was previously referred to as temporal arteritis [8]. The revised diagnostic criteria of GCA include arteritis of the aorta and branching arteries, which can be called large vessel-type GCA [9]. Without the involvement of the temporal arteries, the symptoms of GCA can be vague, such as general fatigue, body weight loss, fever, and systemic muscular pain [9]. Large-vessel GCA can show presentations similar to PMR and can be mistakenly treated for PMR with a low steroid dose. Considering the vagueness in the differentiation of the two diseases, initiating treatment with a low dose of steroids is inevitable.

The clinical course after prednisolone administration should be carefully monitored to differentiate between the two diseases. Initiating a low dose of steroids such as prednisolone (15-20 mg/day) can mitigate the symptoms of both GCA and PMR [10]. The clinical courses of GCA and PMR can differ in the tapering phase of prednisolone treatment. In PMR, tapering of prednisolone is smooth, and its reduction to 10 mg can be accomplished within several months. In contrast, as GCA requires moderate to high doses of prednisolone and sparring drugs for tapering steroids, tapering steroids from 20 mg to a lower dose can fail with a resurgence of inflammatory reactions [11]. This failure is suggestive of GCA without temporal artery inflammation. When treating PMR, the timing of failure requires further investigation, suspecting a large-vessel-type GCA.

For effective diagnosis of large-vessel-type GCA, smooth collaboration among physicians in rural hospitals and clinics is necessary. Failure to taper steroids can be experienced in primary care while treating PMR [10]. The duration from treatment failure to referral to hospitals can be critical for diagnosing and treating large-vessel GCA. Effective collaboration among physicians in rural hospitals and clinics can lead to prompt diagnosis and treatment of large vessel-type GCA [12]. Thus, establishing good relationships among physicians is essential, especially in rural contexts with a lack of healthcare professionals [13]. Effective interprofessional collaboration should be established in the rural healthcare system to diagnose autoimmune diseases.

## Conclusions

This case report describes a large-vessel-type GCA in an older woman in a rural context, mimicking PMR. Effective collaboration between physicians in rural hospitals and clinics is necessary for diagnosing and treating large-vessel GCA. In rural areas, without adequate healthcare professionals, physicians should share their abilities and collaborate smoothly to mitigate delays in consultation and treatment. To effectively treat large-vessel-type GCA, rural general physicians should be familiar with the clinical course of the disease and treatment for comprehensive rural care.

## References

[1] Smith JH, Swanson JW (2014). Giant cell arteritis. Headache.

[2] Nordborg E, Nordborg C (2003). Giant cell arteritis: epidemiological clues to its pathogenesis and an update on its treatment. Rheumatology (Oxford).

[3] Buttgereit F, Dejaco C, Matteson EL, Dasgupta B (2016). Polymyalgia rheumatica and giant cell arteritis: a systematic review. JAMA.

[4] Dejaco C, Brouwer E, Mason JC, Buttgereit F, Matteson EL, Dasgupta B (2017). Giant cell arteritis and polymyalgia rheumatica: current challenges and opportunities. Nat Rev Rheumatol.

[5] Anwar MM, Tariq EF, Khan U, Zaheer M, Ijaz SH (2019). Rheumatoid vasculitis: is it always a late manifestation of rheumatoid arthritis?. Cureus.

[6] Ohta R, Ryu Y, Sano C (2022). Older people’s help-seeking behaviors in rural contexts: a systematic review. Int J Environ Res Public Health.

[7] Stone JH, Tuckwell K, Dimonaco S (2017). Trial of tocilizumab in giant-cell arteritis. N Engl J Med.

[8] Robinette ML, Rao DA, Monach PA (2021). The immunopathology of giant cell arteritis across disease spectra. Front Immunol.

[9] González-Gay MÁ, Ortego-Jurado M, Ercole L, Ortego-Centeno N (2019). Giant cell arteritis: is the clinical spectrum of the disease changing?. BMC Geriatr.

[10] Garvey TD, Koster MJ, Warrington KJ (2021). My treatment approach to giant cell arteritis. Mayo Clin Proc.

[11] Gonzalez-Gay MA, Lopez-Diaz MJ, Barros S (2005). Giant cell arteritis: laboratory tests at the time of diagnosis in a series of 240 patients. Medicine (Baltimore).

[12] Ohta R, Ryu Y, Otani J (2020). Rural physicians’ perceptions about the challenges of participating in interprofessional collaboration: Insights from a focus group study. J Interprof Educ Pract.

[13] Ohta R, Ryu Y, Yoshimura M (2021). Realist evaluation of interprofessional education in primary care through transprofessional role play: what primary care professionals learn together. Educ Prim Care.

